# Secondary cardiac lymphoma presenting with cardiac tamponade and cardiac mass: a case report

**DOI:** 10.1186/s40959-024-00202-8

**Published:** 2024-05-18

**Authors:** Wei Juan Lim, Neerusha Kaisbain, Rafidah Abu Bakar, Hafidz Abd Hadi, Ahmad Khairuddin Mohamed Yusof

**Affiliations:** 1 Institut Jantung Negara (National Heart Institute), 145, Jalan Tun Razak, Wilayah Persekutuan Kuala Lumpur, 50400 Malaysia; 2 Queen Elizabeth II Hospital, Luyang Commercial Centre, Lorong Bersatu, Off, Jalan Damai, Kota Kinabalu, Sabah, 88300 Malaysia

**Keywords:** Cardiac tamponade, Pericardiocentesis, Cardiac lymphoma, Case report

## Abstract

**Background:**

Cardiac tamponade as the presenting manifestation of systemic lymphoma is relatively uncommon. Pericardium is the commonest site of involvement in secondary malignancies with systemic lymphoma involving the heart in 20% of the cases.

**Case presentation:**

We describe a case of a 78-year-old gentleman, who presented with symptoms of new onset cardiac failure, and hemodynamic compromise. An echocardiography revealed cardiac tamponade, necessitating an emergency pericardiocentesis. With the aid of multimodality imaging, he was found to have a right atrioventricular groove mass, widespread lymph node enlargement with bone and peritoneal involvement. Ultimately, a histopathological evaluation revealed a diagnosis of Diffuse Large B Cell Lymphoma (DLBCL).

**Conclusions:**

Our case illustrates that a patient with DLBCL may present with cardiac tamponade as a result of metastasis. This diagnosis, although rare, is likely to be missed, which can cause fatal complications, such as cardiac tamponade, fatal arrhythmias or sudden cardiac death.

**Supplementary Information:**

The online version contains supplementary material available at 10.1186/s40959-024-00202-8.

## Background

The heart is a relatively rare site for the development of tumours [1]. When it occurs, it is more likely to be a secondary cardiac tumor, which may originate from melanoma, lymphoma, lung, breast or renal cancers [1]. Pericardium is the commonest site of involvement in secondary malignancies [1, 2]. Systemic lymphoma may involve the heart in 20% of the cases [3, 4]. Cardiac involvement in these lymphomas usually represents a late manifestation of the disease and this carries a poorer prognosis [5, 6]. They may manifest as pericardial effusion [6]. Nonetheless, patients usually present with extracardiac symptoms instead making the diagnosis cardiac involvement in systemic lymphomas more challenging and more likely to be missed [2, 3]. 

## Case presentation

A previously healthy 78 years old gentleman presented to our emergency department with worsening shortness of breath of two days duration with reduced effort tolerance and palpitation. He denied chest pain, fever or weight loss. His initial blood pressure was 90/60mmHg with a heart rate of 90 beats per minute. Clinical examination revealed a raised jugular venous pressure and a muffled heart sound. Respiratory examination was unremarkable.

**Fig. 1:**
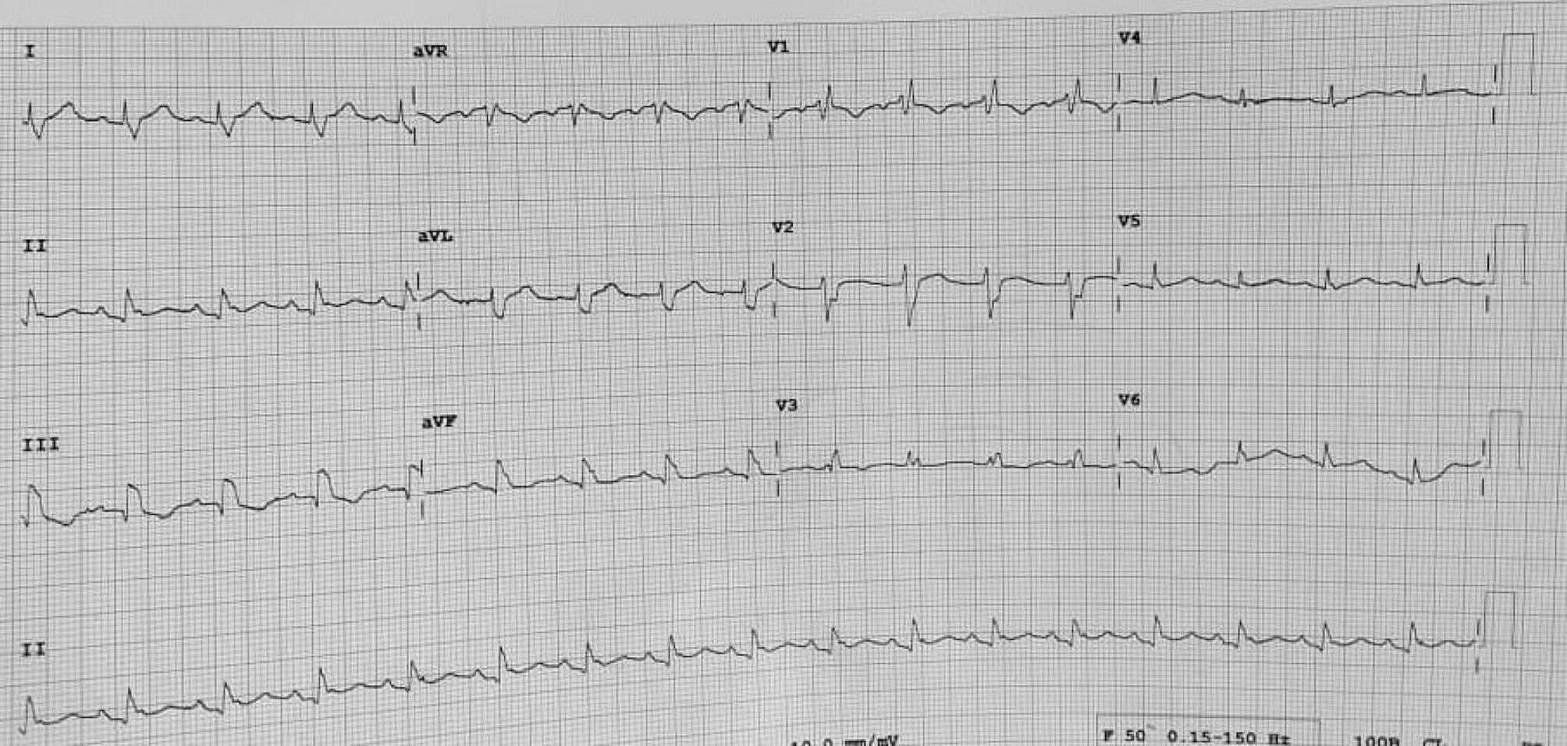
Electrocardiogram showing right bundle branch block

**Fig. 2:**
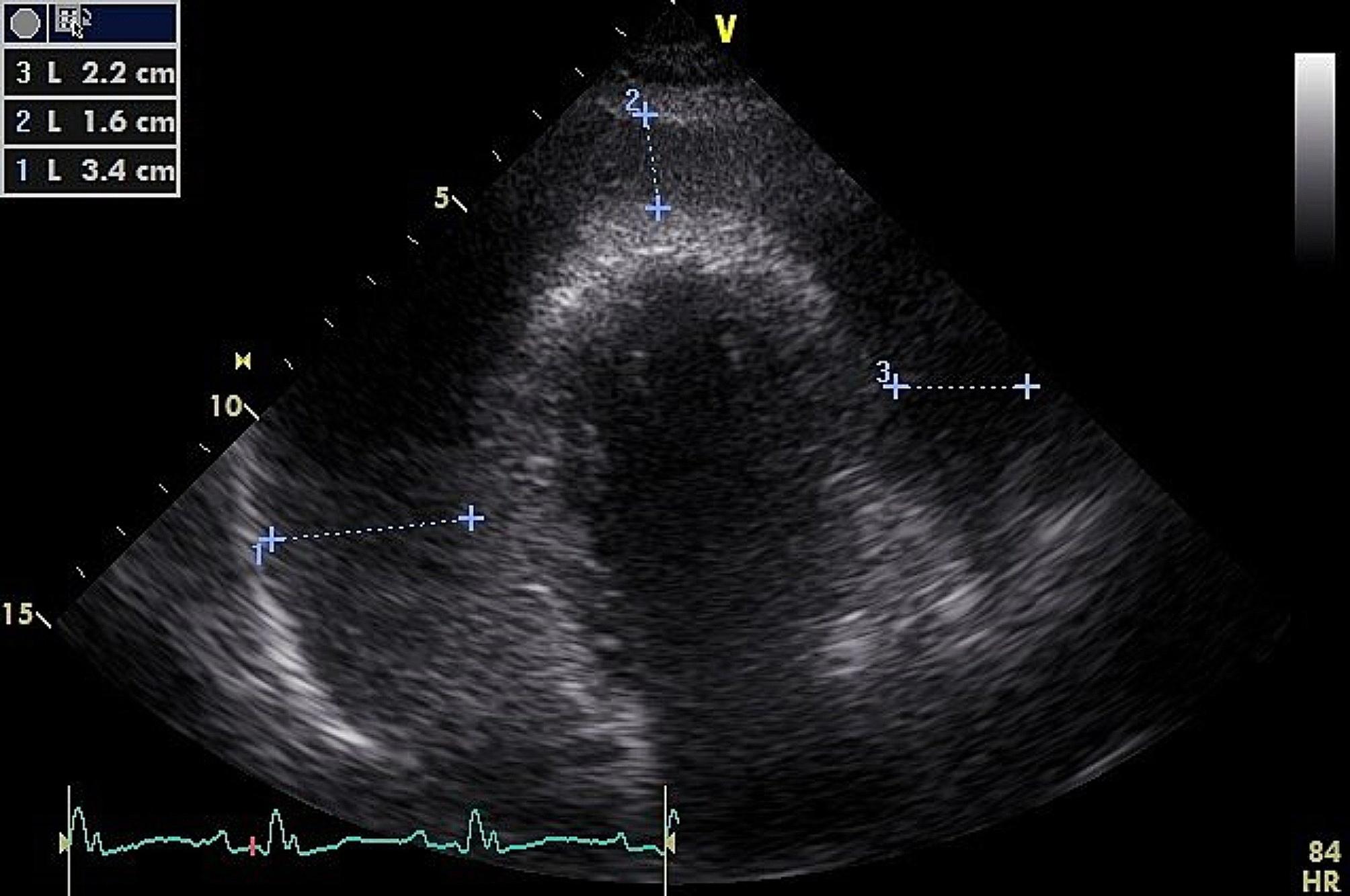
Echocardiogram showing massive pericardial effusion

Electrocardiogram showed right bundle branch block [Figure 1]. A bedside echocardiogram showed global pericardial effusion with evidence of tamponade [Figure 2]. An emergency bedside pericardiocentesis was performed and the pericardial fluid was sent for analysis. Biochemical analysis of the pericardial fluid to serum lactate dehydrogenase (LDH) ratio was 6.9. Pericardial fluid cytology revealed atypical lymphoid cells and hence a contrast enhanced computed tomography (CECT) of thorax, abdomen and pelvis (TAP) was done in active search for malignancy. It showed homogeneously enhancing soft tissue masses in anterior pericardial fat with the most dominant mass encasing the right coronary artery. It was also associated with mediastinal lymphadenopathy and right pulmonary embolism.

**Fig. 3:**
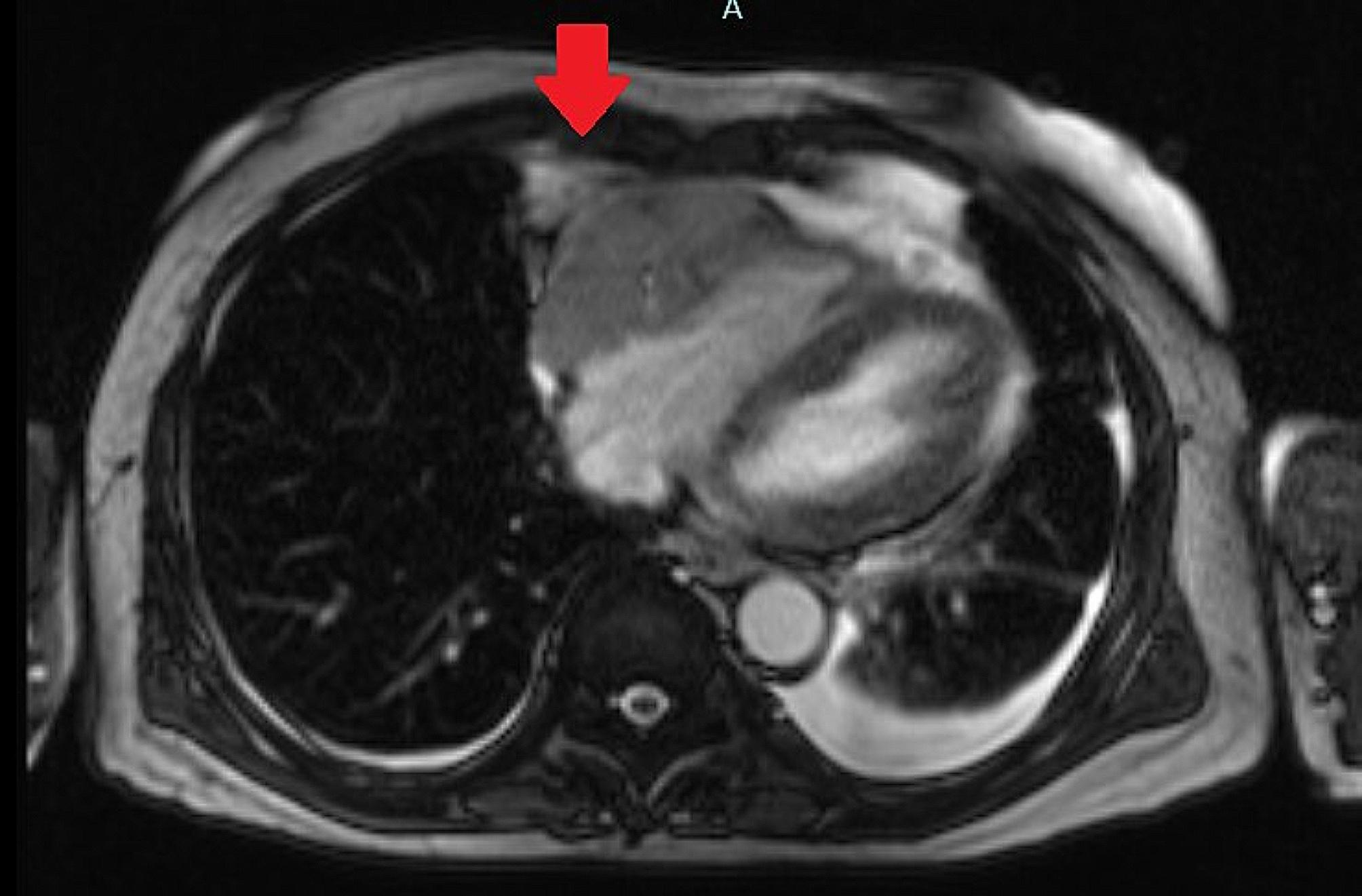
Cardiac magnetic resonance imaging showing mass from right atrioventricular groove

Cardiac magnetic resonance imaging (MRI) confirmed the presence of a mass sized 35 × 61 mm arising from right atrioventricular (AV) groove suggesting the likelihood of a malignant tumour (Fig. 3). Positron Emission Tomography (PET) scan showed metabolically active cardiac mass, likely cardiac lymphoma with widespread lymph node involvement on both sides of diaphragm. In addition, there was peritoneal and bone involvement. A computed tomography (CT) guided biopsy of the mass was performed and the histopathological evaluation of the mass was consistent with diffuse large B cell lymphoma. He developed paroxysmal atrial flutter during his hospital stay.

A total of 1 L of haemoserous pericardial fluid was drained over a course of three days via an indwelling pericardial catheter. A repeated transthoracic echocardiography performed four weeks post pericardiocentesis showed a mild pericardial effusion of size 1.27 cm. The patient was referred to a hematology center for further treatment.

The patient was commenced on dexamethasone and cyclophosphamide while in the hematology center. The patient and family were not keen for intensive chemotherapy and they opted for palliative care. He was discharged with a short course of oral corticosteroids, cyclophosphamide and low molecular weight heparin. He was referred to hospice care.

## Discussion

Cardiac tumours are relatively rare and are divided into primary and secondary cardiac tumours [1]. Secondary cardiac tumors are thirty to forty folds more common as compared to primary cardiac tumours and they usually originate from melanoma, cancers of the lung, breast, kidney, and also lymphoma [1]. The site most commonly involved in secondary cardiac tumors is the pericardium, causing pericardial effusion and occasionally pericardial masses [1]. 

Secondary cardiac lymphoma is more common than primary cardiac lymphoma [3]. 20% of systemic lymphoma has cardiac involvement and it is usually a late manifestation of the disease with poorer prognosis [4, 5, 6]. In an early study by Peterson et al. in 1976, median onset of cardiac involvement is 20 months after an initial diagnosis of lymphoma [7]. As a result, symptoms and swelling outside the heart presents earlier, for an example, painless, superficial lymph node enlargement with systemic symptoms such as fever, night sweats and weight loss, whereas, heart related symptoms are delayed [2, 3]. Thus, symptomatic, massive pericardial effusion as the presenting manifestation of high-grade systemic lymphoma, as seen in our case, is very rare [3]. In contrast, pericardial effusion is more common in Primary Mediastinal Large B Cell Lymphoma (PMBL) patients and is seen in 32% of the cases [8].

Patients with cardiac lymphoma, be it primary or secondary, may present with chest pain, symptoms of cardiac failure and arrhythmias [2]. Our patient presented with dyspnoea, reduced effort tolerance and palpitation. He had paroxysmal atrial flutter, signifying a potential involvement of the conduction system, especially with the tumour being in atrioventricular groove and in close proximity to the conduction pathway [9, 10]. He also presented in tamponade necessitating an emergency pericardiocentesis. As mentioned, massive pericardial effusion as primary manifestation of malignant lymphoma is very rare. Clinically, pulsus paradoxus and Beck’s triad consisting of distant heart sound, raised jugular venous pressure and hypotension signifies cardiac tamponade [5]. However, signs of Beck’s triad, in combination, are only 50% sensitive in picking up this life-threatening condition [5]. 

Electrocardiogram (ECG) is a useful initial screening tool for cardiac tamponade. Patients with cardiac tamponade or massive pericardial effusion may show sinus tachycardia, low voltage QRS complexes, defined as maximum QRS amplitude of < 0.5mV in limb leads and electrical alternans [4]. Electrical alternans means alternating amplitude or axis in QRS complex in any or all leads and it represents swinging heart in pericardial fluid [4]. Echocardiography is an indispensable imaging modality for pericardial effusion [4]. Swinging heart can be appreciated if the volume of the pericardial effusion exceeds 500 ml, but it is uncommon [5]. Other echocardiographic features of cardiac tamponade include, right atrium and right ventricular diastolic collapse, distended inferior vena cava and > 25% respiratory variation in mitral inflow [5]. 

CT scans and Cardiac Magnetic Resonance Imaging (CMR) are helpful tools in characterizing the cardiac mass and severity of pericardial effusion [Table 1].

**Table 1:** Comparison of imaging modalities to study pericardium

Target	Computer Tomography	Magnetic Resonance Imaging
Pericardial thickness	Excellent	Excellent
Pericardial calcifications	Excellent	Poor or not possible
Pericardial inflammation	Good	Excellent
Pericardial effusion	Excellent	Excellent
Transudative effusion	< 10 Hounsfield Units (HU)	Low signal intensity on T1-weighted images
Exudative effusion	20 to 60 Hounsfield Units (HU)	Medium to high signal intensity on T1 sequences
Effusion characteristic	Good	Good
Pericardial mass	Good	Excellent

In our patient, CT scan and CMR revealed the presence of mass in the right atrioventricular groove encasing the right coronary artery with mediastinal lymph node enlargement complicated with pulmonary embolism. Fluorodeoxyglucose Positron Emission Tomography/Computed Tomography (18FDG PET/CT) scanning is more accurate imaging modality for evaluating extension of lymphomas, including evaluating the cardiac masses and detecting extracardiac tumour proliferation and metabolism throughout the body [2, 11]. It is a gold standard imaging method in evaluating disease extension [2]. In our patient, it concluded that the patient has enlarged cervical, mediastinal, abdominal and pelvic lymph nodes with cardiac, bone and peritoneal involvement.

Multimodality imaging must be accompanied by histopathological examination in order to arrive at an accurate diagnosis [12]. Pericardial fluid cytology can be helpful and may show monoclonal lymphocytes, especially in cases where extensive whole-body search does not yield any lymph node or mass for biopsy [3, 11]. Ultimately, tissue diagnosis is indispensable [13]. However, some cardiac lymphomas, especially, primary cardiac lymphoma poses a challenge, as there is a difficulty in obtaining the sample for histopathological evaluation [2]. Fortunately, our patient with secondary cardiac lymphoma had extracardiac mass which was amenable for biopsy. It was in favour of Diffuse Large B Cell Lymphoma (DLBCL), the most common pathological type of secondary cardiac lymphoma [2]. 

Treatment of choice in Non-Hodgkin’s Lymphoma is chemotherapy [14]. Rituximab is frequently used in such lymphomas due to its established efficacy [11]. Pericardial effusions as a result of secondary cardiac lymphoma may respond well with systemic chemotherapy, without the need for local intervention, unlike pericardial effusions occurring as a result of metastatic solid tumours [8]. However, malignant pericardial effusion may recur. In these cases, one may consider treatment with surgical pericardial window, percutaneous balloon pericardiotomy, subxiphoid pericardiotomy and pericardial sclerosis [5]. 

## Conclusion

Our case illustrates that a patient with DLBCL may present with cardiac tamponade as a result of metastasis. This diagnosis, although rare, is likely to be missed, which can cause fatal complications, such as cardiac tamponade, fatal arrhythmias or sudden cardiac death.

### Electronic supplementary material

Below is the link to the electronic supplementary material.

Supplementary Material 1

Supplementary Material 2

## Data Availability

No datasets were generated or analysed during the current study.

## Abbreviations

AV: atrioventricular
CECT: contrast enhanced computed tomography
CMR: Cardiac Magnetic Resonance Imaging
CT: computed tomography
DLBCL: Diffuse Large B Cell Lymphoma
ECG: Electrocardiogram
LDH: lactate dehydrogenase
MRI: magnetic resonance imaging
PET: Positron Emission Tomography
PMBL: Primary Mediastinal Large B Cell Lymphoma
TAP: thorax, abdomen and pelvis
